# Genetic Diversity, Population Structure, and Andean Introgression in Brazilian Common Bean Cultivars after Half a Century of Genetic Breeding

**DOI:** 10.3390/genes11111298

**Published:** 2020-10-30

**Authors:** Caléo Panhoca de Almeida, Jean Fausto de Carvalho Paulino, Sérgio Augusto Morais Carbonell, Alisson Fernando Chiorato, Qijian Song, Valerio Di Vittori, Monica Rodriguez, Roberto Papa, Luciana Lasry Benchimol-Reis

**Affiliations:** 1 Centro de Pesquisa em Recursos Genéticos Vegetais, Instituto Agronômico (IAC), Campinas, São Paulo 13075-630, Brazil; jeanbiotec@gmail.com (J.F.d.C.P.); luciana.reis@sp.gov.br (L.L.B.-R.); 2 Centro de Grãos e Fibras, Instituto Agronômico (IAC), Campinas, São Paulo 13075-630, Brazil; scarbonell@iac.sp.gov.br (S.A.M.C.); afchiorato@iac.sp.gov.br (A.F.C.); 3 Soybean Genomics and Improvement Laboratory, US Department of Agriculture–Agricultural Research Service (USDA-ARS), Beltsville, MD 20705, USA; qijian.song@ars.usda.gov; 4 Dipartimento di Scienze Agrarie, Alimentari ed Ambientali, Università Politecnica dele Marche, 60131 Ancona, Italy; valeriodivittori@gmail.com (V.D.V.); r.papa@staff.univpm.it (R.P.); 5 Max-Planck-Institute of Molecular Plant Physiology, Am Müehlenberg 1, 14476 Potsdam-Golm, Germany; 6 Dipartimento di Agraria, Università degli Studi di Sassari, 07100 Sassari, Italy; mrodrig@uniss.it; 7 Centro per la Cobservazione e Valorizzazione della Biodiversità Vegetale (CBV), Università degli Studi di Sassari, 07040 Alghero, Italy

**Keywords:** *Phaseolus vulgaris* L., common bean, Carioca variety, variability gain, genomic introgression, genome-wide association study

## Abstract

Brazil is the largest consumer and third highest producer of common beans (*Phaseolus vulgaris* L.) worldwide. Since the 1980s, the commercial Carioca variety has been the most consumed in Brazil, followed by Black and Special beans. The present study evaluates genetic diversity and population structure of 185 Brazilian common bean cultivars using 2827 high-quality single-nucleotide polymorphisms (SNPs). The Andean allelic introgression in the Mesoamerican accessions was investigated, and a Carioca panel was tested using an association mapping approach. The results distinguish the Mesoamerican from the Andean accessions, with a prevalence of Mesoamerican accessions (94.6%). When considering the commercial classes, low levels of genetic differentiation were seen, and the Carioca group showed the lowest genetic diversity. However, gain in gene diversity and allelic richness was seen for the modern Carioca cultivars. A set of 1060 ‘diagnostic SNPs’ that show alternative alleles between the pure Mesoamerican and Andean accessions were identified, which allowed the identification of Andean allelic introgression events and shows that there are putative introgression segments in regions enriched with resistance genes. Finally, genome-wide association studies revealed SNPs significantly associated with flowering time, pod maturation, and growth habit, showing that the Carioca Association Panel represents a powerful tool for crop improvements.

## 1. Introduction

The common bean (*Phaseolus vulgaris* L.) belongs to the Fabaceae family, subfamily Faboideae, and genus *Phaseolus* L. This genus has approximately 70 species, of which only five are cultivated. One of these, the common bean, was one of the most successful American crops, and it spread worldwide after the travels of Columbus [1]. Nowadays, the common bean has the largest direct consumption as part of the human diet [2,3,4]. The nutritional value intrinsic to the grain and the potential health benefits define the relevance that this legume has for the human diet, as a source of protein, carbohydrate, fiber, vitamins, and minerals [5].

The global production of dry beans has increased by 77.9% since 2012 (from 6.9 million tons), with a record of 30.4 million tons in 2018 [6]. However, other studies have indicated continued population growth, and therefore, bean consumption is expected to continue to increase in the coming years [3]. In this scenario, Brazil stands out as not only the largest consumer but also the third-largest producer of the common bean, with 10% of annual world production [6,7]. Due to the plasticity of the crop for adapting to different climatic and soil conditions and the range of cultivars available on the market, bean cultivation occurs throughout the year throughout Brazil, with three annual harvests, which guarantees the constant supply of this bean [7].

The common bean originated on the American continent [8,9], from where the species spread to South America, to give rise to two distinct gene pools known as the Mesoamerican and the Andean [10,11]. After subdivision, independent domestication events occurred within each gene pool, making the evolutionary and domestication processes of the common bean a unique model for studies of domestication in plants [9]. The independent domestication processes of these two gene pools are characterized by partial and geographical reproductive barriers, which reflect the strong differentiation between Andean and Mesoamerican accessions in terms of their morphological, biochemical, and molecular traits [8,12,13,14,15,16,17,18].

In Brazil, the common bean varieties that predominated in the consumer market until the 1970s were those with single-colored grain, such as the ‘Roxinho’, ‘Rosinha’, ‘Bolinha’, ‘Preto’, ‘Jalo’, and mainly ‘Manteigão’ types of Andean origin [19]. After the release by the Agronomic Institute (IAC) in Campinas (SP, Brazil) of the cultivar Carioquinha (Carioca Comum) as the first cultivar of the Carioca type in 1971 [20], the scenario of the consumer market underwent drastic changes; today, 49 years after its launch, Carioca represents ~70% of all types of beans consumed in Brazil [21]. The fast acceptance of the new variety was mainly due to the high productive potential that the cultivar Carioquinha exhibited, in addition to resistance to the main diseases. A total of 22 competition trials were conducted between the years 1966 and 1969 involving the Carioquinha cultivar and the other two commercial cultivars mostly harvested in the country, Rosinha G2 and Bico de Ouro. The new cultivar showed superiority in productivity for 15 of the 22 trials, and a higher average yield of approximately ~ 35% in relation to both cultivars, reaching 3490 kg/ha. In addition, the new cultivar had high resistance to bacterial and viral diseases, and to rust [20]. The Carioca variety was derived from the Mesoamerican gene pool, and its cream-colored grain with brown stripes and high productivity [19]. Currently, the main breeding programs in Brazil are focused on improving and obtaining new Carioca cultivars, followed by the Black bean variety, which represents 15% of the varieties consumed in Brazil [21]. Although with lesser efforts, most breeding programs also seek to develop new cultivars for the commercial class of ‘special grains’, such as the varieties ‘Cranberry’, ‘Pinto Beans’, ‘Dark Red Kidney’, ‘Navy’ and ‘Alubia’, which are mainly for export purposes. Unlike Brazil, in Europe, and the U.S., it is found a wide range of bean varieties, with a predominance of accessions of Andean origin in Europe and Mesoamerican origin in the U.S. [15,22,23,24]. In addition to this difference, in Europe, the Andean accessions have a higher level of genetic diversity than the Mesoamerican ones, the opposite of the results observed for American accessions. However, higher rates of hybrid accessions are reported in Europe, and the extensive inter-gene pool hybridization has been identified as the factor responsible for the generation of genetic diversity [15,23].

The *P. vulgaris* species have great genetic variability, mainly for morphological characters such as grain shape, size, and color [21]. However, only a small part of the existing genetic diversity is exploited by breeding programs, mainly due to the difficulty to maintain different phenotypic characteristics that are required from each commercial class. The difficulty is even greater when using breeding strategies that involve crossing different commercial classes and/or wild accessions, which leads to a narrowing of the genetic base of the elite cultivars in comparison to other genotypic groups [25]. Thus, knowing the genetic diversity, studying the population structure, and understanding the relationships of the cultivars within and between commercial classes are fundamental steps for the progression of genetic improvement and for the conservation of genetic diversity of the common beans [24].

Several studies that have analyzed genetic diversity have already been carried out, mainly for morphological [26,27,28,29,30] and molecular [29,31,32,33,34,35] data. Analysis of genetic diversity has demonstrated that there is greater diversity in the Mesoamerican gene pool compared to the Andean gene pool due to a bottleneck that occurred before domestication in the wild populations during the expansion to South America [8,9,18]. An analysis of the diversity and genetic structure of Brazilian landraces using microsatellite markers revealed intermediate genetic diversity in the genotypes in relation to their primary centers of diversity [36]. However, there have not been any studies that have involved genotyping of large numbers of commercial cultivars using technology that guarantees wide coverage of the species genome.

In the current study, a total of 2827 high-quality single-nucleotide polymorphisms (SNPs) that are distributed over the 11 chromosomes of this species were genotyped using SNP Assay technology (Illumina BARCBean6K_3 BeadChip, [37]) with a Brazilian bean diversity panel (BDP) that included 185 accessions from the germplasm bank of the Institute Agronomic—IAC (Campinas, SP—Brazil). Molecular markers were used to: (1) characterize the population structure and genetic diversity of the main cultivars of the Brazilian bean producer market (i.e., Carioca, Black, Special); (2) analyze the diversity gain in terms of the allelic richness and gene diversity of the Carioca variety after almost half a century of genetic breeding; (3) identify Andean allelic introgression in the set of Mesoamerican commercial cultivars; and (4) validate the Carioca accession subset as a diversity panel for a genome-wide association study (GWAS).

## 2. Materials and Methods

### 2.1. Plant Material

For the present study, a total of 185 accessions of commercial cultivars, advanced lines, and some landraces of common bean were selected from the germplasm bank of the IAC, to represent the genetic diversity of the Brazilian common bean. The accessions were selected based on genetic characterization that was carried out by Perseguini et al. [38].

The BDP includes accessions that date from 1963 to 2018 that were released by 11 different genetic breeding institutions in Brazil, both private and governmental. Altogether, 131 accessions belong to the Carioca commercial class, 29 to the Black class, and 25 to the Special class. These include ‘Pinto’, ‘Cranberry’ and ‘Dark Red Kidney’ beans, which were developed for export, and ‘Jalo’, ‘Rosinha’, ‘Alubia’, ‘Mulatinho’, ‘Roxinho’, and ‘Bico de Ouro’ beans, which were marketed before the launch of the Carioca variety. This collection also includes the first Carioca variety that was launched in 1971, Carioquinha (Carioca Comum), which revolutionized the history of beans in Brazil [20]. The cultivar AND 277 that belongs to the Andean gene pool [39] was included in the panel as an Andean genetic pattern.

The Carioca accessions were used as a sub-panel for validation of the GWAS. For the analysis of genetic diversity, these were divided into three groups based on their years of release. The release year of the cultivar BRS-Pérola (1994) was used as a reference, with the first group consisting of cultivars launched from 1971 to 1994 (old Carioca), the second group of cultivars was launched from 1995 to 2018 (modern Carioca), and the third group of lines had unknown release dates (Carioca lines). For association mapping, 10 further Carioca accessions that were not part of the BDP and were previously genotyped were used to increase the size of the GWAS panel, for a total of 141 Carioca accessions. These accessions are not of Brazilian origin or do not have known information, and for that reason, they were not used to the other genetic diversity analyzes. All of the information about the accessions is given in the Supplementary Materials Table S1, including pedigree, commercial class, tegument color, grain size, year of launch, and holding institution.

### 2.2. DNA Extraction, Genotyping and SNP Calling

The DNA of each sample of the BDP was extracted from young leaves (i.e., first trifoliate leaf) collected from five plants of each accession, using the cetyltrimethylammonium bromide (CTAB) protocol described by *Centro Internacional de Mejoramiento de Maíz y Trigo* (International Maize and Wheat Improvement Center; CIMMYT) [40]. The quality of the DNA was confirmed by electrophoresis in 1% agarose, and the quantification carried out using a fluorimeter (Qubit; Thermo Fisher, Waltham, MA, USA). All of the samples were diluted to 50 ng/μL. Genotyping was performed using the BeadChip Illumina technology by BARCBean6K_3 with 5398 SNPs [37]. The quality analysis of the genotypic data was performed using the Genome Studio 2.0 software (Illumina, San Diego, CA, USA), with the elimination of SNPs with *Call Frequency* and *GenTrainScore* < 0.6.

The SNP calling was performed using the TASSEL 5.0 software [41], for which the genotypic matrix was converted into HAPMAP file format, with the reference allele represented by “A”, the alternative allele by “G”, the heterozygous by “R”, and missing data by “N”. SNPs with minor allele frequency < 3%, heterozygosity > 5%, and missing data > 10% were filtered. SNPs with no known position in the genome (i.e., scaffolds) were also removed, and the imputation of “N” *loci* was performed using the Beagle 5.0 software [42].

### 2.3. GWAS of the Carioca Subset

To validate the subset of Carioca cultivars for GWAS, a phenotypic evaluation of 141 Carioca accessions was carried out for days to flowering (DTF), days to pod maturation (DTM), and growth habit (HAB). For this purpose, the accessions were planted at the experimental station of the IAC during the irrigated period (October, 2019). Each experimental plot consisted of a 1-m line with 10 plants, each spaced at 50 cm. The experimental design was as randomized blocks with four replications, and for phenotypic evaluation, only the six central plants of each plot were considered.

For the HAB assessment, four phenotypic classes were considered as proposed by Schoonhove and Pastor-Corrales [43], where: type I (Erect), type II (Semi-erect), type III (Prostrate), and type IV (Climber). The DTF and DTM were evaluated as proposed by the International Centre for Tropical Agriculture - CIAT [44], with DTF as days from the emergence of each seedling until the appearance of the first open floral bud, and DTM as days until the color change of the first pod (i.e., from green to yellow), for at least three plants of the six plants considered for each plot assessed (i.e., 50%).

### 2.4. Statistical Analysis

GenAlEx 6.5 [45] was used to calculate the observed (Ho), expected (He) and unbiased (uHe) heterozygosity, and the Shannon’s Information Index (I) and the number of total private alleles (Npa) using the quality-filtered SNPs. The polymorphic information content (PIC) was calculated using the Cervus 3.0.7 software [46]. The allelic richness (Rs) was estimated using the HP-HARE software [47]; this statistic estimates the number of alleles with correction for population size, as the population size can influence the frequency of alleles [48]. The relative gain or loss of diversity between the old Carioca and the modern Carioca groups was calculated for ΔuHe and ΔRs, according to the formula proposed by Vigouroux et al. [49].

Principal component analysis (PCA) was carried out using the ADE 4 package [50] and visualized graphically using GGPLOT2 [51]. PCA was used to study the relationships among all of the BDP accessions and among just the Mesoamerican accessions. The genetic distances between accessions were estimated by the Nei distances [52] using the POPPR package [53], and grouped using the unweighted pair group method with arithmetic mean (UPGMA). Analysis of molecular variance (AMOVA) with significance tested by 9999 random permutations was used to evaluate the structure of the BDP within the commercial classes and the accessions.

The population structure was evaluated using the Bayesian clustering approach, implemented in the STRUCTURE v2.3.1 software [54], and using the following settings: correlated allelic frequencies; burn-in period of 10,000 and 50,000 Markov Chain Monte Carlo interactions; and grouping (K) ranging from 2 to 11 in 20 independent runs. The ΔK was determined using the STRUCTURE HARVESTER software [55], and the assignment probability of each accession to a certain genetic group (defined as the qi membership coefficient) was determined by alignment of the 20 repetitions of the best K through the CLUMPP method [56], using the CLUMPAK software [57]. The bar graph was generated using the POPHELPER package [58].

Discriminant analysis of the principal component (DAPC) proposed by Jombart et al. [59] and implemented in the ADEGENET v2.1.1 package [60] was used to validate the genetic structure between the commercial classes (i.e., Carioca, Preto, Special). In detail, the genotype matrix was transformed by the PCA into components that can explain most of the genetic variance, which is then used for discriminant analysis, whereby the variance within the commercial class is minimized, and the inter-class variance is maximized. In addition, the *loadingplot* function was used to identify alleles that contribute most to the genetic separation of the three commercial classes.

### 2.5. Andean Introgression into the Mesoamerican Cultivars

To understand the dynamics of the Andean introgression into the Brazilian Mesoamerican cultivars, Bayesian analysis (STRUCTURE) was performed using the parameters previously described, at K = 2, and the analysis was performed separately for each chromosome. Cultivars with 100% Mesoamerican membership coefficients for all of the chromosomes (i.e., pure Mesoamerican) were compared to the 10 Andean cultivars using genotype contrast analysis. This allowed the identification of SNPs with contrasting and diagnostic alleles between the Mesoamerican and Andean cultivar sets. These SNPs are called the ‘diagnostic SNPs’, and are plotted using the MapChart 2.32 software [61]. Using the set of diagnostic SNPs and based on the frequency of the Andean alleles, the genome regions where Andean introgression occurred are identified. Potential genes in linkage disequilibrium (LD) blocks that contained SNPs with an Andean allele frequency >20% were selected for BlastN analysis against the reference genome (*Phaseolus vulgaris* v 2.1; Schmutz et al. [62]), using Jbrowse on Phytozome [63].

### 2.6. Genome-Wide Association Studies

For association mapping, the FarmCPU [64] model in the GAPIT 2.0 package [65] was used. This package explores the multi*locus* mixed model, and performs the analysis in two interactive steps: a fixed-effect model is applied first, followed by a random-effect model, so that both are repeated interactively until no significant SNP is detected. The phenotypic matrix composed for the least squares means of each accession was obtained by restricted maximum likelihood using the JMP 7 software (SAS Institute). To avoid type I errors (i.e., false positives), the structuring matrix was tested using the Bayesian Information Criterion (BIC) test according to Schwarz [66], for a regular mixed linear model [67] with the first five components of the PCA. The *p*-value threshold of each SNP was determined by the resampling method using the function *FarmCPU.P.Threshold*. Each trait was permuted 300 times to break the relationship with the genotypes, and then the random association between all of the SNPs to the phenotype was estimated. The minimum *p*-values obtained among all of the SNPs for the 300 repetitions were recorded, and then the 95% quantile from all of the minimum *p*-values was defined as the *p*-value threshold [68]. Bonferroni [69] threshold method (cut-off, α = 0.01) was also used to test the significance in the Manhattan plot.

To determine the genome window interval used in the BlastN analysis against the reference genome of the SNPs with Andean allelic frequency > 20% and significant SNPs in the GWAS, LD decay was estimated using squared allele-frequency correlation intrachromosomal pairs, with the R package LDcorSV [70], when accounting for the population structure (structure results at K = 2) in the BDP and the relatedness in the Carioca subset (Kinship [71]). The LD decay curves for all of the chromosomes in each panel was explained using the nonlinear model proposed by Hill and Weir [72], as described by Diniz et al. [73].

## 3. Results

### 3.1. Population Structure, General SNP Diversity, and Genetic Relationships

Overall, 2827 high-quality SNPs with polymorphism rates > 3% and well distributed over the 11 chromosomes of the species were filtered from the initial set of BeadChip (BARCBean6K_3, Illumina) and were used for the BDP analysis.

**Figure 1:**
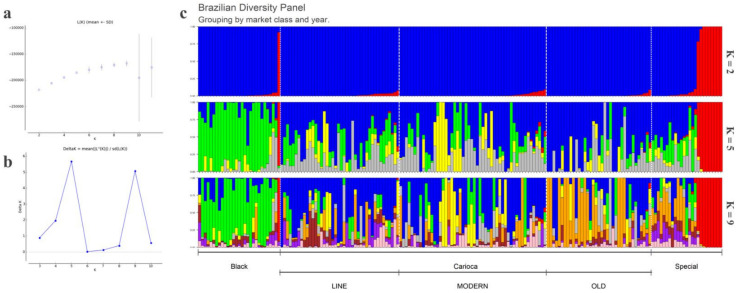
(**a**) Mean values of L (k) for the 20 runs of each K. (**b**) Plot of ΔK for each K value. (**c**) Structure analysis for 185 Brazilian cultivars grouped by commercial class (i.e., Black, Carioca, Special) and year of launch for the Carioca group (i.e., Carioca lines, modern Carioca, old Carioca) for K = 2 (top clustering), K = 5 (middle clustering), and K = 9 (bottom clustering).

As expected, the population structure analysis (Figure 1c, top clustering) evaluated by the Bayesian clustering approach made it possible to clearly distinguish the Mesoamerican accessions (Figure 1c, blue cluster) from the Andean accessions (Figure 1c, red cluster) at K = 2 and showed that the BPD was mainly composed of Mesoamerican cultivars (94.6%). The Andean accessions were restricted to the group of special cultivars, except for cultivar IAC-Diplomata, which belonged to the group of Black beans. Interestingly, no accession was considered as a hybrid (i.e., an admixture between the two gene pools); however, it was possible to identify the Mesoamerican accessions with the highest Andean introgression rate. Cultivars IAC-Tigre, BRS-Cometa and Pintadinho Precoce showed the highest introgression rate, with a probability of being assigned to the Andean pool of 10%, 9.3%, and 9%, respectively. Finally, the AND 277 accession was assigned to the Andean gene pool (q = 1), thus validating its use as an Andean genetic standard.

According to Evanno et al. [74] when followed at K = 2, the data are likely to be structured into five clusters (K = 5) (Figure 1c, middle clustering), as the highest peak was 5 on the Delta K plot (Figure 1a), followed by nine clusters (K = 9) (Figure 1c, bottom clustering). At K = 5, it was possible to distinguish the Black commercial group from most of the others, as seen by the green color in Figure 1c (middle clustering). However, there was no clear structure for the other commercial groups. When considering the presence of nine clusters, in addition to the distinct structure of the Black group, a well-defined group of accessions emerged within the old Carioca group (Figure 1c, orange) that was differentiated from the other Carioca groups (i.e., modern Carioca, Carioca lines). The results for K = 2, K = 5, and K = 9 are given in the Supplementary Materials Table S2.

Considering the division of accessions into both of the gene pools using structure analysis, the Mesoamerican accessions showed higher allelic richness (Rs = 1.52) than the Andean accessions (Rs = 1.36). The same was confirmed using the Shannon’s I and uHe (Table 1). Although the size of both sets was very different, the Rs and uHe parameters were corrected for population size. The Npa of the Mesoamerican accessions (1839) was also greater than for the Andean accessions (422), confirming the higher variability of the Mesoamerican set.

### 3.2. Genetic Relationships and Diversity

**Figure 2:**
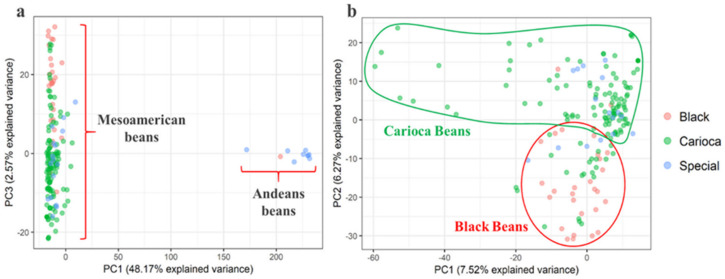
(**a**) Principal component analysis based on the 2827 SNPs for the total set (*n* = 185) (**b**) and on only the Mesoamerican accessions (*n* = 175).

To investigate the relationships between the accessions that make up the BDP, the Nei [52] genetic distance and PCA were estimated. The first component (PC1) explained 48.2% of the variance, and completely separated the Andean from the Mesoamerican accessions, demonstrating the genetic contrast between these two gene pools (Figure 2a); this confirmed the results from the STRUCTURE analysis. A second PCA analysis was performed considering only the Mesoamerican group to provide better resolution of the internal population structure (Figure 2b). Here, PC1 explained 7.5% of the variance, and mainly separated accessions within the Carioca group (Figure 2b, green dots). PC2 explained 6.2% of the variance, and mainly separated the Carioca accessions (Figure 2b, green dots) from the Black accessions (Figure 2b, red dots). The Special accessions were admixed in the PCA analysis, between both the Carioca and the Black groups, and they separated within the group mainly along with the second component (Figure 2b, blue dots).

The PCA and the dendrogram (not shown) indicated the great distance between the Andean and Mesoamerican accessions, although due to the small number of Andean accessions, this was estimated only for the Mesoamerican accessions. Considering the commercial classes, there was no clear genetic differentiation according to the dendrogram shown in the Supplementary Materials Figure S1a. However, there was a trend for the cultivars from the same commercial group to cluster together, as seen mainly for the Carioca accessions. The genetic distances estimated for the three commercial classes showed the closest proximity of the Carioca varieties to the Special varieties, with the Black beans the most distant group genetically (Supplementary Materials Figure S1b).

The accession Oito-Nove, which has a black tegument, showed the greatest genetic distance from the others, while seven landraces (i.e., Cajuri Antigo, Bico de Ouro, Carioca de Cipó, Carioca Guairá, Carioquinha de Produtor, Mourinho, Mulatinho Bico de Ouro) were identical to the first Carioca cultivar released (Carioca Comum). The genetic distance analysis confirmed the pedigree of several cultivars, such as cultivar IAC-Polaco, which grouped close to cultivars Branquinho and IAC-Imperador, which were used as the parental lines for its development, and cultivar IAC-Pyatã, which was closest to cultivar IAC-Akytã, both of which were derived from the same crossing [75].

For the genetic diversity among the commercial classes, the highest was seen for the Special commercial group, for all of the evaluated parameters (i.e., Rs, I, uHe), with an emphasis on uHe (0.40), which was around three times that of the Carioca (0.11) and Black (0.15) groups (Table 1). Although the size of the group represented by the Special accessions was smaller than the others, it was made up of both Andean and Mesoamerican accessions, which was not the case for the Carioca group. Furthermore, the Special group had 89 unique alleles (i.e., private alleles), while the Carioca and Black groups had six and zero unique alleles, respectively. In general, the PIC values ranged from 0.081 to 0.309 (mean, 0.160), with the SNPs ss715641303, ss715641725, ss715649976, ss715647163, ss715647165, and ss715640413 as the most polymorphic among these accessions.

### 3.3. Differentiation among the Commercial Classes

**Figure 3:**
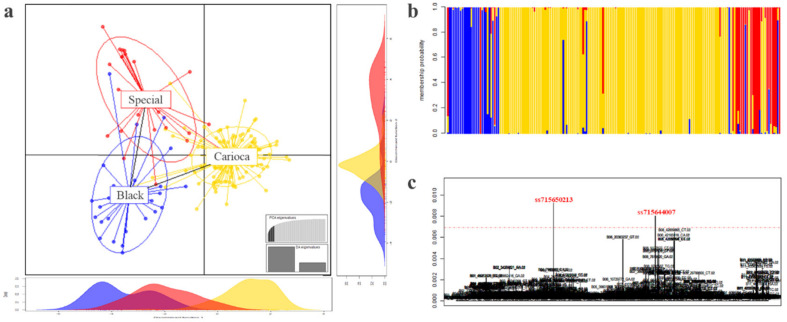
Discriminant analysis of the principal components carried out for the 185 Brazilian common bean cultivars. (**a**) Scatter plot of the two main components grouped by commercial class (colors and ellipses). (**b**) Representation of the *compoplot* function, showing the membership coefficient of each cultivar to each group (i.e., commercial class) ordered according to commercial class (i.e., Black, Carioca, Special), following the scatter plot colors. (**c**) Loading plot generated by the 2827 SNPs, showing the SNPs that contribute most to the separation of the cultivars between the three commercial classes.

The differentiation among the commercial classes was tested using DAPC and AMOVA, and was also used to determine the genetic variation explained by the commercial classes. The DAPC plot (Figure 3a) showed genetic differentiation between the commercial classes, mainly for the Carioca group, which showed the smallest variance within group, as well as the smallest overlap with the other commercial classes. The *compoplot* function (Figure 3b) allowed a better visualization of the accessions belonging to a commercial group that were attributed to another group (Figure 3a; points intersecting an ellipse with a different color). The greatest overlap occurred for the Special group, where six accessions showed coefficients of membership to the group of < 50%, such as cultivars IAPAR-16 and IAPAR-31. These were both classified as Special according to the market class, but had membership coefficients of the Black and Carioca groups of 85% and 80%, respectively. The alleles that mainly contributed to the genetic divergence among the commercial groups were identified through the *loading* function. Polymorphisms at the SNPs ss715650213 (Pv04), ss715645828 (Pv08), and ss715644007 (Pv08) were the most important for distinguishing between the groups (Figure 3c).

The AMOVA showed that the genetic variance restricted with the commercial classes was 23.8% (*p* < 0.0001), with the highest variance restricted between accessions within each class (67%, *p* < 0.0001), with only 8% (*p* < 0.0001) for within accessions, as expected for an autogamous species (Table 2). The *Fst* estimate of the genetic variation between the commercial classes was 0.24.

### 3.4. Diversity Gain of the Carioca Variety

The lowest genetic diversity was seen for the commercial Carioca class (Table 1), and especially for the Shannon’s I (0.19). When only this group was considered, the cultivars launched before 1994 (old Carioca) had lower Rs (1.93), Shannon’s I (0.16), PIC (0.081), and uHe (0.094) than the modern cultivars launched after BRS-Pérola (modern Carioca: Rs = 1.97, I = 0.198, PIC = 0.1, uHe = 0.12). No private alleles were seen for either of the subgroups of old Carioca and modern Carioca; however, accessions from the Carioca lines showed two private alleles. The comparison of these results between the old Carioca and modern Carioca cultivars showed a gain in diversity; less in terms of allelic richness (ΔRs = −0.02), and greater in terms of gene diversity (ΔuHe = −0.25).

### 3.5. Linkage Disequilibrium Decay

**Figure 4:**
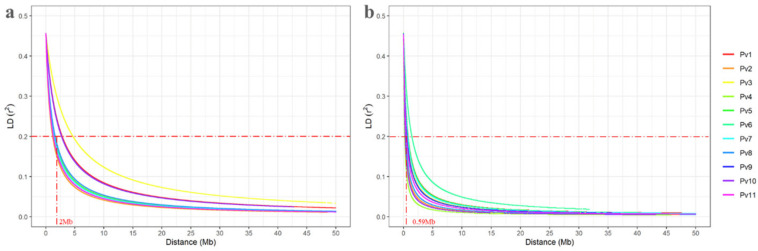
Linkage disequilibrium (LD) decay determined by the LD measurements (r^2^) against the distance between SNPs (Mb) for the 11 chromosomes (Pv) adjusted according to the model proposed by Hill and Weir (1988). (**a**) Brazilian diversity panel controlled for population structure. (**b**) Carioca panel controlled for relatedness. The horizontal and vertical lines represent the standard LD decay thresholds (r^2^) and distances (Mb), respectively.

As expected, the LD decay curve showed a clear tendency for mean LD values (r^2^) to decrease as the distance between SNPs increased, for both sets analyzed (Figure 4). However, the decay of the Carioca panel (Figure 4b; mean r^2^ = 0.046) was considerably greater than for the BDP (Figure 4a; mean r^2^ = 0.11). The chromosomes Pv01 (mean r^2^ = 0.12), Pv03 (mean r^2^ = 0.12) and Pv11 (mean r^2^ = 0.11) of the BDP, and Pv06 (mean r^2^ = 0.08) of the Carioca panel had the highest mean r^2^ values. When considering the standard LD decay threshold (r^2^ = 0.2), the mean distances for all the chromosomes of the BDP and the Carioca panel were 2.00 Mb and 0.59 Mb, respectively.

Considering the small size of the bean genome (~587 Mb) and the distance of 0.59 Mb for the decay of LD to 0.2 for the Carioca panel, the minimum number of SNPs required for good genome coverage and satisfactory GWAS data was 995, just over half of the number used in the present study (1927 SNPs).

### 3.6. Andean Introgression

**Figure 5:**
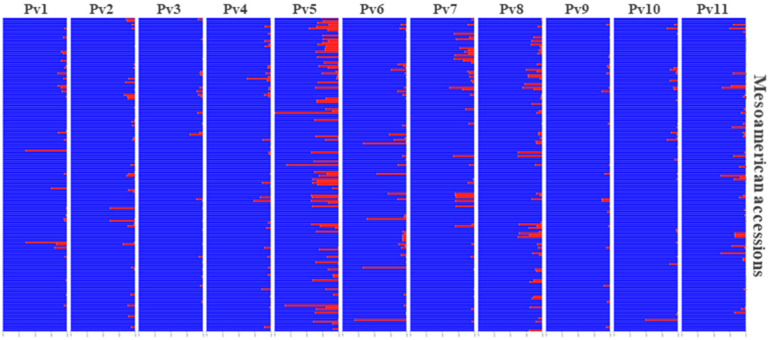
Structure analysis for each chromosome individually plotted for K = 2 to identify events of chromosome introgression of the Andean gene pool (red cluster) into the Mesoamerican accessions (*n* = 175). Each plot represents one chromosome (as indicated), and the bars represent the Mesoamerican cultivars.

**Figure 6:**
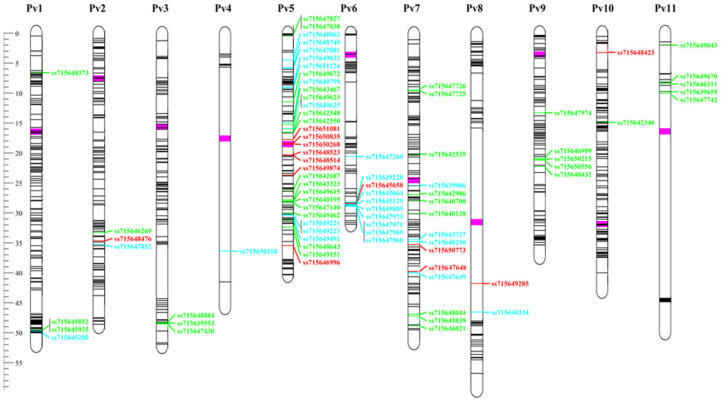
Distribution of the 1060 diagnostic SNPs in the common bean genome. The 82 SNPs with Andean allele frequency from 11% to 15%, 16% to 19%, and > 20% in the Mesoamerican pool are in green, blue, and red, respectively. SNPs with Andean allele frequency < 10% are in black. The centromeric region of each chromosome is in pink.

STRUCTURE analyses were performed for each of the chromosomes individually (Figure 5). These data showed that allelic introgression occurred predominantly on chromosome Pv05 for almost all the cultivars. This highlighted the Pintadinho Precoce landrace, which had 99% of allelic Andean introgression for chromosome Pv05. The chromosomes with the lowest introgression rates were Pv03 and Pv09, and 22 cultivars were identified with total absence of Andean introgression for all chromosomes (i.e., pure Mesoamerican). These pure Mesoamerican cultivars were used for genotype comparisons against the 10 Andean cultivars, through which two sets of polymorphic SNPs between the Andean and Mesoamerican groups were identified. The first set of 552 SNPs shows the “GG” genotype for all of the Andean accessions, and the “AA” genotype for all of the Mesoamerican accessions, while the reverse was seen for the second set of 508 SNPs (i.e., “AA” genotype, Andean; “GG” genotype, Mesoamerican). In general, 1060 SNPs were monomorphic within each gene pool, but polymorphic between the Mesoamerican and Andean pools, and were thus defined as the ‘diagnostic SNPs’ (Figure 6). These SNPs showed satisfactory distribution throughout the genome, except for chromosomes Pv04 and Pv11 (5, 15 SNPs each, respectively).

Using the set of diagnostic SNPs, it was possible to identify the Andean alleles that showed the highest frequencies across all the Mesoamerican cultivars. Considering only the SNPs with an Andean frequency >10%, introgression was observed for all the chromosomes (89 SNPs). For the SNPs that showed introgression >20% (maximum minor allele frequency, 29%), seven SNPs were identified on chromosome Pv05, two on Pv07, and only one on Pv02, Pv06, Pv08, and Pv10. This thus showed the greatest introgression levels for chromosome Pv05, which agreed with the STRUCTURE results (Figure 5). Five SNPs on chromosome Pv05 were located in a centromeric region and spanned over <2.7 Mb, as very close to each other and in strong LD (r^2^ > 0.8), thus showing a large Andean allele segment in this region (SNPs: ss715651081, ss715650835, ss715650268, ss715648523, ss715648514).

The BlastN analysis of the SNPs with the highest Andean allele occurrence in the reference genome of the species revealed two SNPs located within a genic sequence. In general, most of the SNPs were in regions enriched with genes, such as ss715645658 on chromosome Pv06, located inside a 2-Mb window containing 261 genes. Genetic annotation of the genes showed distinctive functions, such as a zinc finger protein (Phvul.002G182400), a transcription factor (Phvul.005G119400), an RNA helicase protein (Phvul.006G179000), a growth-regulating factor (Phvul.007G222300), a vacuolar iron transporter protein (Phvul.010G021600), and others. However, genes related to disease resistance were the most common among these, such as those encoding the leucine-rich repeat protein kinase family protein LLR (e.g., Phvul.006G174400), the auxin-responsive GH3 protein (e.g., Phvul.005G089200), a pectin lyase-like superfamily protein (Phvul.002G188700), and others. SNP ss715648423 on Pv10 was associated with the largest number of resistance genes (i.e., 44 resistance R genes: TIR-NBS-LRR class).

The genes that were located within 2 Mb windows centered on the SNPs with the highest Andean allele occurrence are given in the Supplementary Materials Table S3, as well as the respective genetic annotations, positions, and distances.

### 3.7. Genome Wide Association Studies

After quality control and SNP calling, 1927 high-quality SNPs were selected for association mapping on the panel of 141 Carioca accessions that were phenotypically evaluated for DTF, DTM, and HAB. The ANOVA results showed significance differences for cultivars, and repetition for all traits, except for DTF (Table 3). The coefficient of variation was relatively low, particularly for DTM (2.7%). Heritability in the broad sense (h^2^) was considered high for all the traits. Shapiro-Wilk tests revealed nonnormality just for HAB, mainly because the Carioca accessions are elite germplasm bank genotypes that have undergone several selection cycles, such that none of the evaluated accessions has growth habit type 4 (i.e., climbers). As expected, Pearson’s correlation showed a significant positive correlation between DTF and DTM (r = 0.50), which showed that the plants that flower first tend to be earlier for pod maturation. A significant positive correlation was also seen between HAB and DTF (r = 0.17) and HAB and DTM (r = 0.20), indicating that erect accessions tend to be more precocious.

To determine whether correction for population structure should be applied to the GWAS model, PCA was performed for the genetic matrix, and the BIC test was performed on the first five components, which together explained 27.6% of the variance. According to Schwarz (1978), the highest BIC reveals the best number of covariates for the model. For all traits, no PCs were required (not shown). This was confirmed by the PCA (Supplementary Materials Figure S2), where no definite sub-structures were present, and the first component explained only 8% of the variance.

**Figure 7:**
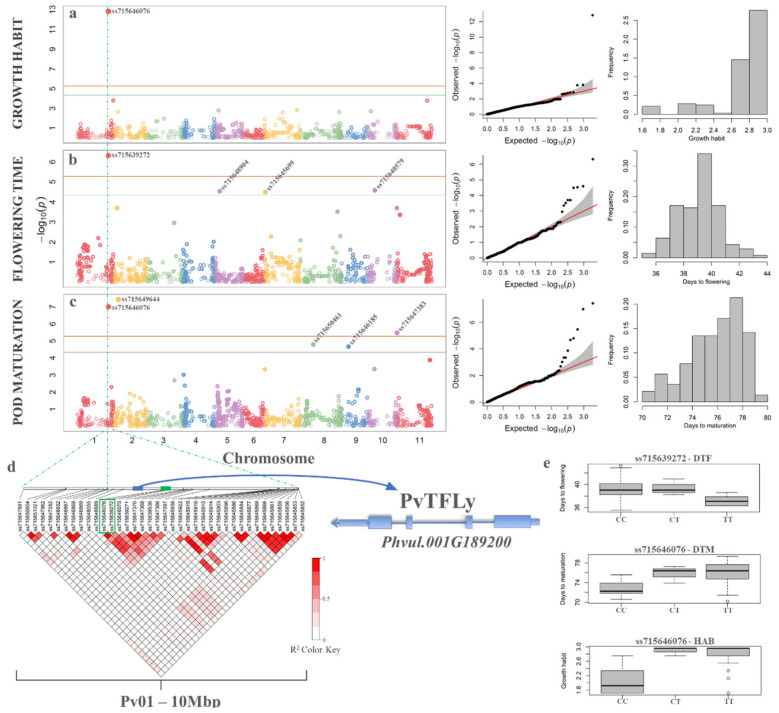
Genome-wide association study of the Carioca association panel. (**a**–**c**) Manhattan plots showing the association between the SNP markers and the growth habit (**a**), flowering time (**b**) and pod maturation (**c**). The green dotted line passes through the SNPs with the lowest *p*-values for all of the trait associations on Pv01. (**d**) Linkage disequilibrium (r^2^) heatmap in the 10 Mb region surrounding the most significant SNPs (ss715646076, ss715639272) associated with all of the traits, which were located 389 kb and 299 kb, respectively, upstream of the *PvTFL1y* gene (Phuvl.001G189200). (**e**) Boxplots illustrating the relationships between alleles and phenotypes (as indicated) for the significant SNPs on Pv01.

The GWAS results indicate that SNP ss715646076 that is located on chromosome Pv01 (position 45,156,837 bp) showed a significant marker phenotype association for HAB (Figure 7a) and DTM (Figure 7c), and was the only one associated with HAB (Figure 7a). A second SNP (ss715639272, position 45,246,824 bp) in LD (r^2^ = 1) with SNP ss715646076 was the only one significantly associated with DTF (Figure 7b). The distance between ss715646076 and ss715639272 is 0.09 Mb, and both are in a region composed of a block of eight SNPs in LD (Figure 7d). Two other SNPs showed significant association for DTM, one on Pv02 (ss715649644; position 7,303,124 bp), and the other on Pv10 (ss715647383; position 43,057,291 bp).

For the SNP associated with HAB, the accessions with two copies of the reference allele “C” tended to be more erect than the accessions with two copies of the alternative allele “T”, or the heterozygote “C/T” (Figure 7e). A similar trend was seen for the association with DTM, with genotypes “C/C” earlier than genotypes “T/T” or “C/T”.

The BlastN analysis of SNP ss715646076 against the Andean common bean reference genome (Phytozome v2.1; Schmutz et al. [62]) showed that this SNP is located on the third intron of the Phvul.001G192700 (functional annotation: sister chromatid cohesion 1 protein) gene, which is ~389 kb downstream of the *Terminal Flower 1* gene (*PvTFL1y*; Phvul.001G189200). The other SNP on Pv01 (ss715639272) is located between genes Phvul.001G193600 (upstream) and Phvul.001G193700 (downstream) at ~100 kb from each of these genes, both without functional annotation. SNP ss715649644 had the lowest *p*-value for DTM and is located ~299 kb upstream of Phvul.002G062000 (functional annotation: L-lactate dehydrogenase). The third SNP significantly associated with DTM, ss715647383, is located at ~1.9 kb from Phvul.010G150400 (without functional annotation).

## 4. Discussion

Our study shows the potential usefulness in crop genetics studies of the Brazilian panel, which comprises 185 accessions of common bean (dried beans) that were selected tof represent the genetic diversity of commercial cultivars, advanced lines, and landraces in Brazil. It is a panel that includes several commercial varieties that harbor high molecular diversity. Here, we have characterized both the molecular and the phenotypic diversity of the BDP panel based on 2827 SNPs and on three phenotypic traits of the Carioca commercial type, which represents a subset of 141 accessions that was useful to highlight the structure of the diversity of the Brazilian genotypes and their potential for association studies.

We showed two contrasting genetic groups that correspond to the Andean and Mesoamerican gene pools, with the Mesoamerican predominant in the BDP panel. This result was expected for Brazil, where most of the germplasm has a Mesoamerican origin [36,76]. This is similar to what has been seen in the U.S. and Canada, where the predominance of the Mesoamerican gene pool also occurs, as indicated by the most consumed commercial varieties of the ‘Navy’, ‘Black’, ‘Small white’, ‘Great northern’, ‘Pink’, ‘Small red’, and ‘Pinto’ beans [24,77]. However, this is different from other countries, such as Europe, where several studies have reported predominance of the Andean gene pool [15,78,79,80,81].

The Andean accessions show lower levels of genetic diversity (uHe = 0.10; Rs = 1.36; I = 0.15) than the Mesoamerican accessions (uHe = 0.12; Rs = 1.52; I = 0.21) for all of the parameters calculated. Several other studies have reported higher genetic diversity of the Mesoamerican gene pool, when comparing both improved commercial accessions [9,62,82] and wild genotypes [4,14]. More specifically in Brazil, which is in geographical proximity to the Andes, the predominance of Mesoamerican accessions is of interest, which according to Burle et al. [36], might be due to the prevalence of Mesoamerican introduction [10] and to the similarities in the climatic conditions between Brazil and the Mesoamerican region. Valdisser et al. [76] suggested that this might explain the lower diversity of the Andean accessions from Brazil, in addition to the historical events of domestication in the evolution of the common bean [3,9,62,83].

### 4.1. Andean Introgression into Brazilian Mesoamerican Cultivars

Unlike other studies [17,22,23,38,76,84], no admixed or hybrid accessions (i.e., a similar mixture of Andean and Mesoamerican genetic pools) were identified in the accessions evaluated here. However, several Mesoamerican accessions were identified as carrying some introgression from the Andean pool, according to the population structure. This indicates some hybridization between the gene pools, with several *loci* showing introgression from the Andean into the Mesoamerican gene pool. This might be due to the use of Andean accessions as donor parents in some Brazilian breeding programs. The pedigree data support this, where some Andean parents were included in the crosses in the development of Mesoamerican cultivars, such as IAC-Alvorada, BRS-Talismã, IAC-Milênio, IPA9, IAC-Tigre, and IAC-Galante [85,86,87,88]. The Black bean cultivar IAC-Diplomata is another example; although it was classified as an Andean accession by the population structure analysis, it was obtained from the same cross that originated the Carioca cultivar IAC-Alvorada, which is classified as Mesoamerican [85]. The distribution of the introgression peak within the Carioca genotypes might highlight the signature of selection [18] that occurred during the Carioca breeding programs in Brazil. This is also supported by the knowledge that historically, in the Brazil breeding programs, Andean donor parents were used as a source of disease resistance. Examples include the use of Andean resistance gene sources in areas where Mesoamerican isolates were predominant [89]. AND 277 is an example of an Andean cultivar that was used in breeding programs because of its resistance to angular leaf spot [90], powdery mildew [91], anthracnose [92], and rust [93], and it has been widely used in Brazil and southern Africa [89]. The use of Andean genotypes as donor parents for Mesoamerican breeding might be of great interest, as it is not particularly common in other countries.

At least one resistance-related gene was found in the genetic annotation for the diagnostic SNPs selected, with the R genes the most frequent, such as for SNP ss715648423 on chromosome Pv10, in a region where 41 R genes are located. The R genes are involved in resistance to several diseases, including those of bacterial, viral, and fungal origin, and also in resistance to insects and nematodes. Most of the R genes encode intracellular proteins with a conserved central nucleotide-binding domain (also known as NB-ARC), and a more variable C-terminal leucine-rich repeat domain [94,95]. In addition, several genetic mapping studies have reported *loci* of resistance to various diseases close to these diagnostic SNPs. Oblessuc et al. [96] identified ALS10.1^UC^ on Pv10 as the quantitative trait *loci* (QTL) with the greatest angular leaf spot resistance. Oblessuc et al. [97] performed fine mapping of the same QTL and identified the IAC54 marker in the QTL interval, which is 41.1 kb downstream from the diagnostic SNP s715648423. Wu et al. [98] identified the marker NSSR260 as associated with resistance to common bacterial blight on Pv10, located 492 kb from diagnostic SNP s715648423.

### 4.2. Diversity, Genetic Structuring, and Differentiation in Commercial Classes

Different from other countries, in Brazil, 85% of common bean consumption is of only two commercial varieties: Carioca and Black beans (Pereira et al., 2019). Both belong to the Mesoamerican gene pool, as also demonstrated in the present study. However, the greatest genetic diversity and allelic richness were observed for the Special group (uHe = 0.40; Rs = 1.99; I = 0.57), which can be explained by the presence of both Andean and Mesoamerican accessions in this group. For the Carioca (uHe = 0.11; Rs = 1.65; I = 0.19) and Black (uHe = 0.15; Rs = 1.89; I = 0.25) groups, both had a relatively low genetic diversity, and very similar values, although the genetic diversity of the Black group was a little higher. Similar genetic diversity was demonstrated by Delfini et al. [29] and Cardoso et al. [99] for Black and Carioca cultivars. Both studies reported higher values of diversity for Black cultivars compared to Cariocas cultivars. Rodriguez et al. [17] analyzed a domesticated bean set and also reported close genetic diversities for these (He   =  0.15).

In the present study, the Black group also had a genetic structure that was clearly differentiated from the other groups, as based on the Bayesian analysis and in relation to the restricted genetic variance among the commercial classes (23.8%), with this value very similar to that reported by Delfini et al. [29] (i.e., 29%). On the other hand, the low *Fst* (0.24) and the high genetic variance among the accessions (67.5%) showed that the genetic differentiation is not well defined. This was supported by the DAPC, in which overlap was seen among the commercial groups. In the case of the varieties such as Carioca beans, for which the seed coat appearance and color are extremely important for the consumer market, the use of nonCarioca accessions in the breeding process is particularly challenging due to the ease with which the grain can lose its ideotype. This might explain the higher proportions of nonCariocas accessions with the Carioca background in the *compoplot* illustrated in Figure 3b, rather than the inverse.

### 4.3. Gain in Genetic Diversity after Almost Half a Century of Genetic Breeding

After the Carioca variety was accepted, which was a balance between several common bean varieties, the Brazilian consumer market changed drastically, with Carioca beans becoming the most consumed variety in the country [21]. The first Carioca genotype was created from a bean crop of the type Chumbinho Opaco (a variety widely consumed in the mid-1960s), which probably originated from a natural mutation [100]. With the launch of the first Carioca cultivar in 1971 [20], breeding programs started using the cultivars available up to that time to improve the Carioca variety. This explains the presence of a prevalent cluster in the old Carioca group, illustrated in Figure 1c as the orange cluster (K = 9), which was also present in the Special group. Some examples here include the cultivars Rosinha G2, IAC-Aroana 80, and Tupi, all of which have a membership coefficient for this orange cluster >46%. These Special cultivars were used before the launch of the first Carioca variety, and indeed, probably served as a basis for the beginning of the breeding process of the Carioca variety.

Normally, during common bean genetic breeding processes, the breeders exploit only a small part of the existing genetic diversity, due to the intensive use of elite parent lines for the crosses, with the aim being to increase the chance of success in breeding for a given trait [21]. Thus, the commercial cultivars tend to have a narrow genetic base compared to the wild genotypes [25]. This process was accentuated for Carioca, which arose from a single plant a few decades ago, and due to the complexity of the grain color pattern, the use of different accessions in the breeding process is costly. However, although the Carioca group has the lowest genetic diversity, the subgroup of modern Carioca cultivars showed greater diversity than the old Carioca cultivars, which has resulted in a gain in genetic diversity over the last 24 years of genetic breeding, both in terms of allelic richness (ΔRs = 2%) and genetic diversity (ΔuHe = 25%).

In the present study, the gain in genetic variability shows the success of the Brazilian breeding programs in terms of increasing the variability of the new Carioca cultivars. In particular, the greater increase in the genetic diversity (ΔuHe) when compared to the allelic diversity (Rs) for cultivars launched after 1994 shows that the variety improvement process is still recent and that the expected heterozygosity has been the main source of variation for the selection process over this short time. Indeed, the number of alleles is more sensitive to changes in the population size and bottlenecks than to the expected heterozygosity [101,102,103].

### 4.4. Validation of the Carioca Diversity Subset in Genome Wide Association Studies

Genome-wide association studies were performed for HAB, DTF, and DTM in the sub-panel of the 141 Carioca common bean accessions. This showed two SNPs (i.e., ss715646076, ss715639272) in LD and ~89.9 kb apart that were significantly associated with all the traits evaluated. Among the 1927 SNPs that we used from the BARCBean6K_3 chip, the significant SNPs ss715646076 and ss715639272 were the closest to the *PvTFL1y* gene (Phvul.001G189200), which controls the determinate HAB in common bean [104], and which is located in the *fin locus* on chromosome Pv01 that was previously identified by Koinange et al. [105].

Cichy et al. [82] mapped the same SNP ss715639272 for HAB in a panel of 396 accessions, and Kamfwa et al. [106] used an Andean diversity panel to identify a significant SNP (SS715646578) for DTF and DTM that was 2.2 Mb from the haplotype block that was in LD with the associated SNPs in the present study. Kamfwa et al. [106] also reported that the Phvul.001G221100 gene in the same region is a candidate in the control of precocity, which encodes for the phytochrome A (PHYA). For SNPs ss715649644 (Pv02) and ss715647383 (Pv10) that were associated with DTM in the present study, Nascimento et al. [107] evaluated a panel of 80 common bean Brazilian cultivars in four environments and other two seasons, and they reported two SNPs mapped to a distance of 2.9 Mb and 1.9 Mb from SNPs ss715649644 and ss715647383, respectively, one where the significance was greatest, and the another that explained the greatest proportion of phenotypic variance for precocity. Genetic annotation showed that SNP ss715647383 is 0.57 Mb upstream of the Phvul.010G142900 gene, which is responsible for the synthesis of the early flowering 3 protein, which acts in the initiation of the flowering time [108,109].

## 5. Conclusions

This is the first study on genetic diversity, population structure, and gain in genetic variability that examines a large number of Brazilian accessions. The 2827 molecular markers genotyped here has provided insights into the levels of diversity of the three main commercial classes of beans in Brazil. A strong population structure was identified for the Black bean group and a high degree of genetic and allelic diversity for the Special bean group. Among the commercial classes, Carioca beans showed the lowest diversity. However, over almost half a century of Brazilian breeding programs since the release of Carioca, a significant increase in genetic diversity has been developed. It was also possible to identify the introgression of Andean alleles into the Brazilian Mesoamerican background, which resulted from the selection of novel sources of resistance to the main crop diseases. The Carioca bean panel is shown to be a powerful tool for selection scans and GWAS, which underlines the value of the Carioca association panel for crop improvement.

## Figures and Tables

**Table 1:** Genetic diversity estimated for the Brazilian diversity panel using 2827 single nucleotide polymorphisms (SNPs), considering the genetic pools and the commercial class divisions.

Group	No.	N_pa_	I	Rs	H_o_	H_e_	uHe	PIC
Bean diversity panel	185	-	0.317	-	0.018	0.181	0.182	0.160
Special	25	89	0.573	1.990	0.019	0.403	0.404	0.309
Black	29	0	0.257	1.890	0.018	0.152	0.151	0.128
Carioca	131	6	0.199	1.650	0.018	0.115	0.114	0.099
Modern Carioca	53	0	0.198	1.970	0.017	0.117	0.118	0.100
Old Carioca	36	0	0.163	1.930	0.023	0.095	0.094	0.081
Carioca lines	42	2	0.200	1.980	0.015	0.118	0.117	0.101
Andean	10	422	0.152	1.360	0.002	0.099	0.100	0.080
Mesoamerican	175	1839	0.211	1.520	0.019	0.121	0.122	0.105

No., number of individuals; N_pa_, number of total private alleles; I, Shannon’s Information Index; R_s_, allelic richness; H_o_, observed heterozygosity; H_e_, expected heterozygosity; uHe, unbiased expected heterozygosity; PIC, polymorphic information content.

**Table 2:** Analysis of molecular variance (AMOVA) for the 185 Brazilian cultivars for two genetic levels: commercial classes and cultivars.

Variance Source	df	SS	Variance	
			Component	(%)
Between commercial classes	2	12,889.0	71.3	23.86 *
Between cultivars	182	78,172.9	201.9	67.52 *
Within cultivars	185	4761.0	25.7	8.60 *
Total	369	95,822.9	299.00	100

* *p* > 0.05 (significance test with 9.999 permutations); df, degrees of freedom; SS, sum of squares.

**Table 3:** Variance analysis (ANOVA) for the traits of flowering time, pod maturation, and growth habit for the Carioca panel (*n =* 141), demonstrated by the mean squares.

Trait	Cultivar	Repetition	Residual	CV	h^2^	S-P
Flowering time	14.89 ***	6.06 ^ns^	3.44	4.75%	77.97%	0.07 ^ns^
Pod maturation	25.69 ***	23.79 ***	4.45	2.78%	83.15%	0.51 ^ns^
Growth habit	0.58 ***	1.28 ***	0.93	11.04%	83.09%	0.0001 ***
df	140	3	420			

***, *p* <0.001; ^ns^, not significant (F tests); CV, coefficient of variation; h^2^, heritability in the broad sense; S-P, Shapiro-Wilk normality test; df, degrees of freedom.

## Supplementary Materials

The following are available online at https://www.mdpi.com/2073-4425/11/11/1298/s1, Figure S1: Genetic distance of Nei (1978) estimated for 175 Mesoamerican accessions of the bean diversity panel using 2827 single-nucleotide polymorphisms clustered by UPGMA at the level of accessions (**a**) and population (**b**), Figure S2: Principal component analysis for the 1927 SNPs of the Carioca panel plotted in three-dimensions showing the absence of sub-structures, Table S1: Details of the 185 cultivars: gene pool, commercial classification, year of released, grain size, the institution of origin, genealogy, and genotypic matrix, Table S2: Coefficient of participation from the STRUCTURE analysis of the 185 accessions, for K = 2, K = 5, and K = 9, Table S3: Gene annotation for the results located within a 2-Mb window that contains the diagnostic SNPs with Andean allelic introgression >20% (i.e., minor allele frequency) in the Mesoamerican cultivars, with the disease resistance genes summarized.
