# Supplementary figures and images for: Genetic Diversity, Population Structure, and Andean Introgression in Brazilian Common Bean Cultivars after Half a Century of Genetic Breeding
Source: Genes (Basel). 2020 Oct 30;11(11):1298. doi: 10.3390/genes11111298 (PMC7694079; doi:10.3390/genes11111298)

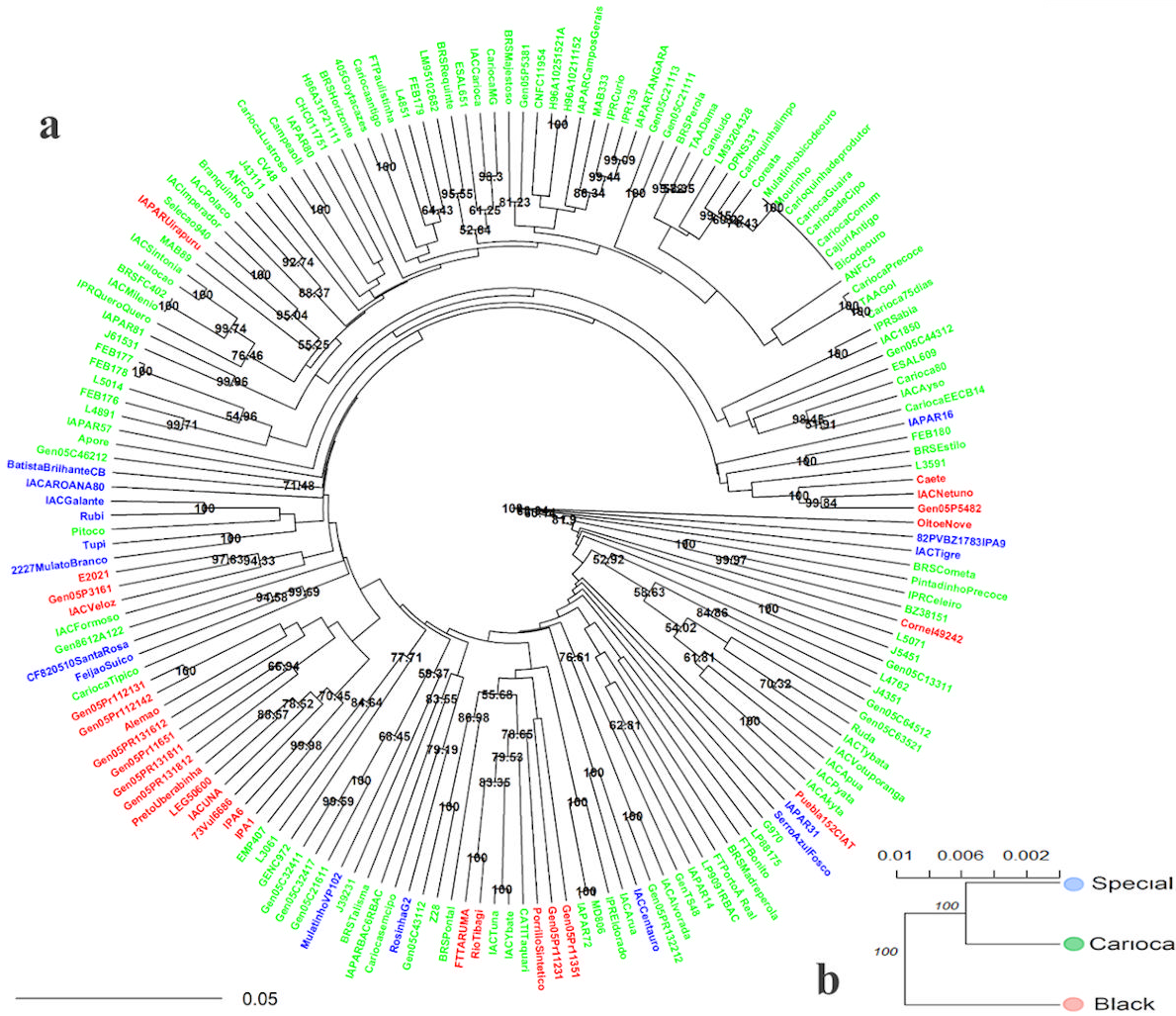

Supplement: Supplementary file 1 [file genes-11-01298-s001.zip › Figure S1.tif]

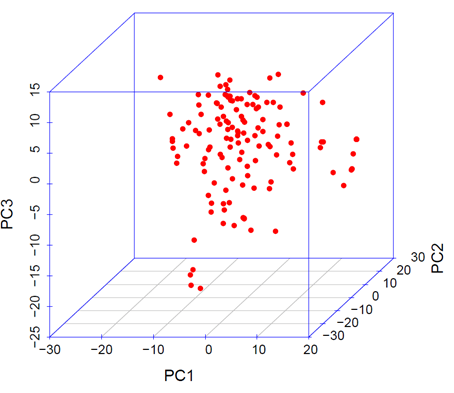

Supplement: Supplementary file 1 [file genes-11-01298-s001.zip › Figure S2.tif]
